# Fannyhessea vaginae causing bacteraemia and vertebral osteomyelitis: first report of invasive disease in a male

**DOI:** 10.1099/acmi.0.000785.v3

**Published:** 2024-04-24

**Authors:** Victoria Jordan, Ayesha Akram, Robert Pickles, Alyssa Arnold, Syeda Naqvi

**Affiliations:** 1 Department of Microbiology, NSW Health Pathology, John Hunter Hospital, New Lambton Heights, NSW, Australia; 2 School of Medicine and Public Health, University of Newcastle, Callaghan, NSW, Australia; 3 Department of Infectious Diseases, John Hunter Hospital, New Lambton Heights, NSW, Australia; 4 Department of General Medicine, John Hunter Hospital, New Lambton Heights, NSW, Australia

**Keywords:** anaerobic urinary tract infection, *Fannyhessea vaginae*, vertebral osteomyelitis

## Abstract

**Introduction.***Fannyhessea vaginae* (formerly *Atopobium vaginae*) is an anaerobic organism commonly associated with female genital flora, with rare cases of invasive disease reported in females.

**Case report.** We discuss the case of an 81-year-old male who presented with an acute history of back pain and signs of urinary tract infection in the context of intermittent self-urinary catheterisation. Multiple blood cultures grew *Fannyhessea vaginae* with a later finding of lumbar vertebral osteomyelitis as the cause of back pain. Treatment was commenced with ampicillin, later switched to ceftriaxone, with improvement of acute signs of infection.

**Conclusion.** Gram-positive anaerobic organisms including *Fannyhessea vaginae* are possibly under-recognised causes of urinary tract particularly in older males. These bacteria may prove challenging to grow in standard protocols for urine culture; anaerobic or extended incubation could be considered particularly in complicated cases of urinary tract infection without an identifiable pathogen.

## Data Summary

No new data were generated in this study.

## Introduction

*Fannyhessea vaginae* (formerly *Atopobium vaginae*) is an anaerobic Gram-positive coccus most frequently isolated from the female genital tract in association with bacterial vaginosis [1,2]. In recent years, there have been several reports of invasive disease with *Fannyhessea vaginae* occurring in females, generally related to gynaecological conditions or surgery [3]. We discuss the first case, to our knowledge, of *Fannyhessea vaginae* bacteraemia and vertebral osteomyelitis in a male.

## Case presentation

An 81-year-old male was admitted for investigation and management of acute severe low back pain that had been progressing over the course of 4 days. He had a background of hypertension, coronary artery bypass surgery, cerebrovascular disease, osteoarthritis, and a right-sided total hip replacement. He had also been self-catheterising twice daily for the preceding 5 years. Regular medications included aspirin, metoprolol, atorvastatin, amlodipine, and perindopril. He was a retired bricklayer who lived at home with his wife. He consumed up to 60 grams of alcohol daily and was a distant ex-smoker. On review, he was afebrile with right lower abdominal tenderness and marked low back pain limiting mobility. Investigations showed leucocytes and nitrites on urine analysis and a significantly elevated C-reactive protein (CRP) to 80 mg l^−1^. In the context of urinary catheterisation, lower abdominal tenderness and leukocytes on urinalysis, urinary tract infection was thought likely; he was commenced on intravenous ampicillin and gentamicin as well as analgesia for the back pain. By day four his CRP had increased further to 319 mg l^−1^ and he still had ongoing severe back pain.

Two separate blood culture sets obtained on admission, both containing BD BACTEC aerobic and anaerobic plus bottles (Becton Dickinson, NJ, USA) subsequently returned positive for Gram-positive cocci. They grew only in the anaerobic bottle at 57 and 69 h respectively, incubated in the BD BACTEC FX automated instrument. Reflex subcultures were set up on non-selective agar incubated both aerobically and anaerobically at 37 °C for 48 h, with growth only occurring on the anaerobic plate. This growth was confidently identified by MALDI Biotyper (Bruker, MA, USA) as *Fannyhessea vaginae* with a top log score of >2.3. The patient was continued on ampicillin pending further results. Susceptibility testing was performed by E-test (bioMérieux, USA) on Brucella blood agar in anaerobic conditions with results summarised in Table 1; Minimum Inhibitory Concentration (MIC) to penicillin was low at 0.50 mg l^−1^ and conversely very high to metronidazole at >256 mg l^−1^, though clinical breakpoints were not available to interpret further. Formal urine microscopy showed 1100×10^6^ leucocytes and growth of *E. coli*. Of note, the urine culture was incubated for 48 h in aerobic conditions only, in accordance with the standard operating procedure of the laboratory, which is insufficient to exclude growth of strict anaerobes including *Fannyhessea vaginae*.

**Table 1.** Antimicrobial susceptibility testing results

Antibiotic	MIC (mg l^−1^) E-test
Clindamycin	<0.02
Metronidazole	>256
Penicillin	0.50
Vancomycin	1.50

**Fig. 1.**
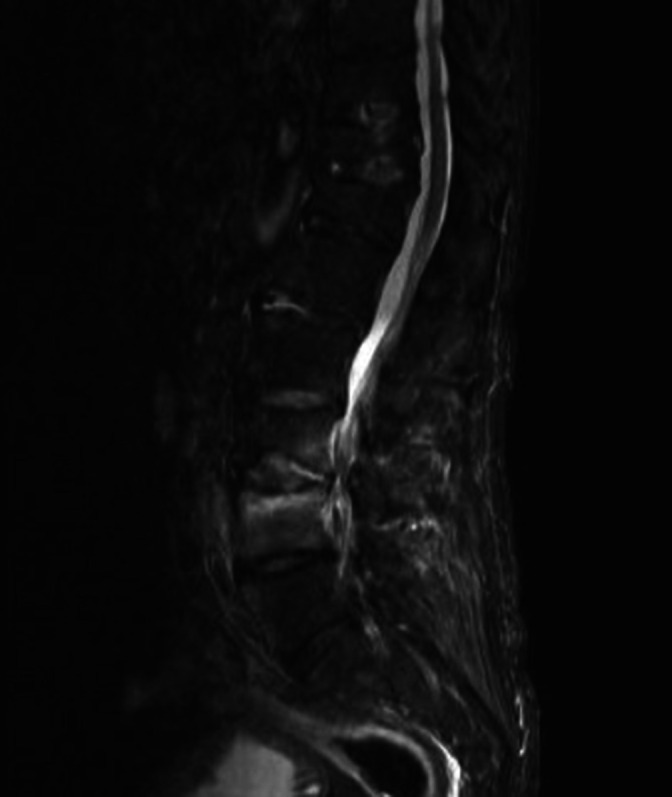
Sagittal MRI STIR sequence of the lumbosacral spine showing hyperintensity in L4 vertebral body and L3/4 disc space.

In view of ongoing back pain, magnetic resonance imaging (MRI) of the spine was performed, which demonstrated L3/4 discitis with osteomyelitis of the L4 vertebral body (Fig. 1). On day 14 of antimicrobial therapy with ampicillin, an imaging-guided biopsy of the L3/4 intervertebral disc was undertaken, and while there were profuse polymorphs on microscopy, there was no growth nor detection of bacterial DNA on 16S rRNA sequencing. The clinical course was then complicated by the onset of delirium prompting the discovery of an enhancing right occipital lobe mass (21×13×11mm) on MRI brain which was found by histopathological examination to be consistent with grade three astrocytoma (anaplastic astrocytoma) rather than an infectious complication. Antibiotics were switched to ceftriaxone during investigation of this lesion, for enhanced central nervous system penetration to cover the possibility of abscess, and 8 weeks of IV therapy were completed with this. The patient’s delirium and inflammatory markers improved, however unfortunately due to residual back pain and deconditioning resulting in functional decline, he required transfer to a residential aged care facility on discharge. A progress CT spine after treatment showed sclerotic changes of the affected L3/4 level without the previously seen enhancement or any new lesions. Further treatment of the astrocytoma was not pursued according to the patient’s wishes.

## Discussion

*Fannyhessea vaginae* is a Gram-positive, obligate anaerobic bacteria of the family *Atopobiaceae* within the order *Coriobacteriales*, and appears as cocci or coccobacilli in pairs or short chains [1,4, 5]. It is fastidious, non-motile, catalase-negative [1] and non-spore forming [2,4]. After originally being described as *Atopobium vaginae* in 1999, phylogenetic analysis prompted reclassification of the organism to the new genus *Fannyhessea* in 2018 of which *F. vaginae* is the type organism [1,6]. It is predominantly found in the female genital tract, constituting normal flora in up to 25 % of women [2,7], however it is more commonly found in women with bacterial vaginosis (BV) often in combination with overgrowth of other BV-associated organisms, particularly *Gardnerella vaginalis,* with which it can form synergistic biofilms [2]. *Fannyhessea vaginae* has also been recognised as a urinary tract colonising organism [7,9].

While historically the urinary tract has been considered sterile, the lower urinary tract is likely colonised by complex populations of bacteria [8]. In healthy adult women, these bacteria can include genera found in BV, including *Fannyhessea*, *Gardnerella*, and *Prevotella* species; similar organisms may be found in sexually experienced males, specifically those who have vaginal sex [8,9]. For example, 7.2–11.4 % of men may have positive urinary tract cultures for *Gardnerella* sp. [10]. When seen in clinical disease, these organisms are usually associated with genitourinary infections in females. However, case reports have been described particularly for *Gardnerella* sp. causing severe infections beyond the genitourinary tract, such as bacteraemia, septic arthritis, and empyema [11].

Similar to *Gardnerella,* invasive disease with *Fannyhessea vaginae* has been reported mostly in females [4,5, 12,14]. The few reported cases of isolated bacteraemia have originated from the female genitourinary tract, including intrapartum, post-hysterectomy and post-chorionic villi sampling [4,5, 12, 13]. Other manifestations have also been seen: in 2018 a case of tricuspid valve endocarditis was reported in an 18-year-old, female, type one diabetic who presented with a sepsis-like presentation. The patient described lancing a vaginal cyst 2 weeks prior to presentation [3]. In 2021, Kolakowska *et al*. described the case of an otherwise healthy 44-year-old woman with L5/S1 discitis, with discovertebral samples growing *Fannyhessea vaginae* as well as *Gardnerella vaginalis*, *Peptostreptococcus indolicus*, and *Prevotella amnii* [15]. While the organisms suggested a genitourinary origin, no other disease was found, and it was hypothesized that infection may have developed following a mucosal injury occurring with sexual intercourse [15].

Our patient unexpectedly grew *Fannyhessea* in the bloodstream, complicated by lower lumbar vertebral osteomyelitis. While the isolation of this organism in the blood cultures suggested a potential genitourinary origin, we could not establish this link due to the inability to grow the organism in the urine cultures. We hypothesised that our patient probably acquired *Fannyhessea* from sexual intercourse and may have subsequently developed invasive infection following a mucosal injury during self-catheterisation, though unfortunately a sexual history was not obtained prior to discharge as it had not been relevant to the immediate presenting illness. A similar case has been reported in the literature in a female patient, a 77-year-old woman who had a periprosthetic right hip infection with repeated growth of *Fannyhessea vaginae* from the joint [16]. She had a concurrent urinary tract infection (UTI) with growth of *Escherichia coli* but *Fannyhessea* was not isolated from the urinary culture [16].

Overall, Gram-positive anaerobic organisms are an under-diagnosed cause of UTI [17], for example *Actinotignum schaali*, an increasingly recognised cause of UTI particularly in elderly males [18]. Failure to isolate anaerobic organisms, including *Fannyhessea* sp*.* and *Actinotignum schaali,* from urine cultures could be attributed to lack of routine anaerobic culture [17,18] and, given that growth of these organisms may be slow [19], the relatively short incubation duration. In our laboratory for example, the primary culture plate is incubated for up to 48 h only in ambient air; as a step to isolate some of these anaerobic or fastidious organisms, if there is significant pyuria without growth after this interval, the sample undergoes repeat subculture onto anaerobic agar and chocolate agar, with incubation occurring in anaerobic and CO_2_-enriched conditions respectively for 48 h. However, if there has already been growth such as in our case (even if non-significant, e.g. skin flora, low quantity of enteric organisms) this does not occur. The literature suggests that in specific cases, such as chronic infection where a causative organism has not been established in patients who are elderly, immunosuppressed, or diabetic, incubation of urine cultures in an anaerobic environment should be considered [17]. The optimal duration for anaerobic incubation of urine samples in this context needs to be clarified, however given the relatively slow growth of these organisms [19], 48 h may be suboptimal. This may be an under-recognised consideration for laboratories when developing urine processing protocols.

*Fannyhessea vaginae* usually has low MICs to penicillins (0.008–<0.25 µg ml^−1^) and clindamycin (<0.016 µg ml^−1^), and variable, often higher MICs to metronidazole (2–>256 µg ml^−1^) [20]; Petrina *et al*. found a median metronidazole MIC of 64 µg ml^−1^ [21]. Successful treatment courses for invasive disease have included clindamycin alone, clindamycin in combination with metronidazole, amoxicillin-clavulanate and amoxicillin [3,4, 15, 16]. However, clinical breakpoints have not been determined specifically for *Fannyhessea* sp*.* previously. The European Committee on Antimicrobial Susceptibility Testing (EUCAST) provides clinical breakpoints for some Gram-positive anaerobes, namely *Cutibacterium acnes*, *Clostridium perfringens* and *Clostridioides difficile*, however these cannot be applied to other organisms [22]. The Clinical and Laboratory Standards Institute (CLSI) does provide breakpoints for Gram-positive anaerobes as a group, for ampicillin, β-lactam combination agents, clindamycin, carbapenems and metronidazole, however with the proviso that many breakpoints for anaerobic organisms were devised prior to routine use of pharmacokinetic-pharmacodynamic data in establishing breakpoints, and that ‘on some occasions, only limited clinical data were used to establish breakpoints’ [23,24]. Our patient completed approximately 8 weeks of IV therapy with ampicillin initially then ceftriaxone. While he had improvement in acute symptoms, inflammatory markers, and radiology, he had ongoing back pain, with other factors, including his age, comorbidities, prolonged hospital stay and brain tumour likely also contributing to poor functional outcome.

## Conclusion

*Fannyhessea vaginae* is commonly isolated from the female genitourinary tract but can also colonise the male genitourinary tract. It has been rarely associated with bacteraemia, endocarditis and bone and joint infections [3,9, 16]. More data is needed to inform optimal treatment and clinical breakpoints for antimicrobial therapy. *Fannyhessea vaginae*, and anaerobic organisms in general, are likely an under-recognised source of urinary tract infection and laboratories should consider primary or adjunctive anaerobic incubation of urine samples in complicated cases without an identifiable pathogen [17].

## Abbreviations

CT: computed tomography
IV: intravenous
MIC: minimum inhibitory concentration
MRI: magnetic resonance imaging
UTI: urinary tract infection
